# Enhanced Cardioprotection by Human Endometrium Mesenchymal Stem Cells Driven by Exosomal MicroRNA‐21

**DOI:** 10.5966/sctm.2015-0386

**Published:** 2016-08-29

**Authors:** Kan Wang, Zhi Jiang, Keith A. Webster, Jinghai Chen, Hengxun Hu, Yu Zhou, Jing Zhao, Lihan Wang, Yingchao Wang, Zhiwei Zhong, Cheng Ni, Qingju Li, Charlie Xiang, Ling Zhang, Rongrong Wu, Wei Zhu, Hong Yu, Xinyang Hu, Jian'an Wang

**Affiliations:** ^1^ Department of Cardiology, Second Affiliated Hospital, School of Medicine, Zhejiang University, Hangzhou, People's Republic of China; ^2^ Cardiovascular Key Laboratory of Zhejiang Province, Hangzhou, People's Republic of China; ^3^ Department of Cardiology, Guizhou Provincial People's Hospital, Guiyang, People's Republic of China; ^4^ Vascular Biology Institute, Miller School of Medicine, University of Miami, Miami, Florida, USA; ^5^ The Institute of Translational Medicine, Zhejiang University, Hangzhou, People's Republic of China; ^6^ Zhejiang‐California International Nanosystems Institute, Zhejiang University, Hangzhou, People's Republic of China

**Keywords:** Mesenchymal stem cells, Endometrium, Bone marrow, Adipose, Myocardial infarction, Cell therapy

## Abstract

Our group recently reported positive therapeutic benefit of human endometrium‐derived mesenchymal stem cells (EnMSCs) delivered to infarcted rat myocardium, an effect that correlated with enhanced secretion of protective cytokines and growth factors compared with parallel cultures of human bone marrow MSCs (BMMSCs). To define more precisely the molecular mechanisms of EnMSC therapy, in the present study, we assessed in parallel the paracrine and therapeutic properties of MSCs derived from endometrium, bone marrow, and adipose tissues in a rat model of myocardial infarction (MI). EnMSCs, BMMSCs, and adipose‐derived MSCs (AdMSCs) were characterized by fluorescence‐activated cell sorting (FACS). Paracrine and cytoprotective actions were assessed in vitro by coculture with neonatal cardiomyocytes and human umbilical vein endothelial cells. A rat MI model was used to compare cell therapy by intramyocardial injection of BMMSCs, AdMSCs, and EnMSCs. We found that EnMSCs conferred superior cardioprotection relative to BMMSCs or AdMSCs and supported enhanced microvessel density. Inhibitor studies indicated that the enhanced paracrine actions of EnMSCs were mediated by secreted exosomes. Analyses of exosomal microRNAs (miRs) by miR array and quantitative polymerase chain reaction revealed that miR‐21 expression was selectively enhanced in exosomes derived from EnMSCs. Selective antagonism of miR‐21 by anti‐miR treatment abolished the antiapoptotic and angiogenic effects of EnMSCs with parallel effects on phosphatase and tensin homolog (PTEN), a miR‐21 target and downstream Akt. The results of the present study confirm the superior cardioprotection by EnMSCs relative to BMMSCs or AdMSCs and implicates miR‐21 as a potential mediator of EnMSC therapy by enhancing cell survival through the PTEN/Akt pathway. The endometrium might be a preferential source of MSCs for cardiovascular cell therapy. Stem Cells Translational Medicine *2017;6:209–222*

**Significance Statement**

The present report describes the functional and mechanistic comparisons of mesenchymal stem cells (MSCs) from three different sources (bone marrow, adipose, and endometrium) as therapeutic agents for the treatment of cardiovascular disease. MSCs from the endometrium have markedly superior therapeutic potential relative to the other cell types, properties that can be explained by the paracrine actions mediated by exosomal microRNAs, in particular microRNA‐21, with downstream effects on cell survival and angiogenesis. The findings are important from a clinical perspective because the endometrium could be a preferential source of MSCs for cardiovascular cell therapy.

## Introduction

Stem cell infusion and intramyocardial injection remain promising approaches for the treatment of coronary artery disease, myocardial infarction (MI), and heart failure 1. Mesenchymal stem cells (MSCs) are potential off‐the‐shelf reagents for such therapy, although their full potential has not yet been achieved, and clinical studies have suggested that bone marrow MSCs (BMMSCs) confer only mild and, in some cases, negligible effects on heart function and infarct size 2 3 4 5. Endometrium‐derived MSCs (EnMSCs) can be conveniently isolated from menstrual blood and can be expanded to create almost infinite supplies 6. Our group recently reported the enhanced therapeutic properties of transplanted EnMSCs, including preservation of cardiac function, decreased infarct size, and ameliorated cardiac fibrosis, mediated primarily by paracrine effects in a rat model of MI 7, 8. Because the tissue origin of MSCs can critically determine the paracrine activity and therapeutic potential 5, 9, it is important to implement comparative studies in parallel to identify the optimal sources for clinical application.

Exosomes are cell‐derived microvesicles that are released from the plasma membrane into the extracellular space, where they play key roles in intercellular signaling 10. Exosome communications can involve the transfer of proteins, cytokines, lipids, mRNAs, ribosomal RNAs, and microRNAs (miRs) 9, 11. Recently, exosomes have been shown to play key roles in the paracrine actions of MSCs and can be central mediators of MSC cytoprotection in the contexts of both myocardial infarction and ischemia/reperfusion injury 12.

In the present study, we compared paracrine functions, in vivo therapy, and exosomal profiles of human EnMSCs, BMMSCs, and adipose‐derived MSCs (AdMSCs). We report the superior actions of EnMSCs in a rat MI model relative to BMMSCs and AdMSCs that correlated with enhanced paracrine actions and higher expression of exosomal miR‐21. The results are consistent with a role for miR‐21 by downregulation of phosphatase and tensin homolog (PTEN) and enhancement of Akt survival kinase activity in the superior actions of EnMSCs in this model.

## Materials and Methods

### Cell Culture

Human BMMSCs and EnMSCs were obtained from S‐Evans Biosciences (Hangzhou, China, http://www.sebio.net.cn). Human AdMSCs were purchased from Cyagen Bioscience (Guangzhou, China, http://www.cyagen.com). Human umbilical vein endothelial cells (HUVECs) were provided by AllCells (Shanghai, China, http://www.allcells.com). The cells were maintained in Dulbecco's modified Eagle's medium (DMEM; Thermo Fisher Scientific, Waltham, MA, http://www.thermofisher.com) with low glucose, supplemented with 10% fetal bovine serum (FBS; Thermo Fisher Scientific) at 37°C in a humidified atmosphere with 5% CO_2_.

### Flow Cytometry

MSCs at passages 6–8 were characterized for surface markers using a FACS Canto II Flow Cytometer (BD Biosciences, Franklin Lakes, NJ, http://www/bdbiosciences.com). In brief, 1 × 10^6^ cells suspended in 100 μl of phosphate‐buffered saline (PBS) with 1% FBS were stained with a panel of antibodies: fluorescein isothiocyanate (FITC)‐CD45, phycoerythrin (PE)‐CD29, allophycocyanin‐CD90, PE‐CD34, PE‐CD105, PE‐CD166, PE‐CD117, and isotype‐matched control for 1 hour. The cells were washed twice with PBS, and at least 10,000 cells per tube were acquired for each test. Surface marker expression was quantified using FACSCount II software (BD Biosciences).

### Preparation of Conditioned Medium

MSCs were cultured to 90% confluence in complete medium. Then, the media were replaced with DMEM containing 2% FBS for an additional 48 hours of incubation. The medium was collected, centrifuged at 2000*g* for 30 minutes, and stored at −80°C.

### Isolation of Neonatal Rat Ventricular Cardiomyocytes

Neonatal rat cardiomyocytes (CMs) were isolated and cultured as described previously 13. In brief, hearts from 1‐day‐old Sprague‐Dawley pups were minced and digested with 0.1% trypsin (Thermo Fisher Scientific). The dispersed cells were cultured with 10% FBS‐supplemented high glucose DMEM (glucose concentration, 4 g/l) containing 100 μmol/l 5′‐bromo‐2′‐deoxyuridine (Sigma‐Aldrich, St. Louis, MO, http://www.sigmaaldrich.com) for 90 minutes at 37°C with 5% CO_2_. Nonadherent cells were collected and seeded onto 24‐well plates (2.5 × 10^5^ cells per well) or 6‐well plates (1 × 10^6^ cells per well; Corning, Tewksbury, MA, http://www/corning.com). The culture medium was replaced 48 hours after seeding. CMs were ready for experimentation after 72 hours of incubation.

### Terminal Deoxynucleotidyl Transferase dUPT Nick‐End Labeling

Terminal deoxynucleotidyl transferase dUTP nick‐end labeling (TUNEL) assays were performed in accordance with the manufacturer's protocol (Roche Applied Science, Indianapolis, IN, http://www.roche_applied_science.com). In brief, the cells or samples were fixed in 4% paraformaldehyde and permeabilized with 0.2% Triton X‐100 for 10 minutes. Nucleotide and TdT enzyme mixture was added to the cells or samples for 1 hour of incubation at 37°C. The nuclei were stained with 4′,6‐diamidino‐2‐phenylindole (DAPI). The percentage of apoptotic nuclei per total CM nuclei was calculated as the index of apoptosis. Images were taken at five randomly selected fields in the border zone of the ischemic myocardium in each sample or per well.

### Immunostaining

Cells and heart samples were fixed with 4% paraformaldehyde for 10 minutes and permeabilized with 0.2% Triton. After 1 hour of blocking by 10% bovine serum albumin (BSA), the cells or tissue samples were incubated with primary antibodies as follows: anti‐troponin I (Abcam, Cambridge, MA, http://www.abcam.com). Sections or cells were washed with PBS‐0.05% Tween (PBST) followed by incubation of secondary antibody (fluorescein Isothiocyanate [FITC]‐conjugated; Abcam). After 1 hour of incubation at 37°C, cell nuclei were stained with DAPI. Images were obtained by fluorescence microscope (Leica Microsystems, Wetzlar, Germany, http://www.leica-microsystems.com) at five randomly selected fields for each sample or well and the mean positive/negative numbers calculated.

### Tube Formation Assay

For tube formation assays, 96‐well plates were precoated with 50 μl of growth factor‐reduced Matrigel (BD Biosciences) for 30 minutes at 37°C. HUVECs were seeded at a density of 20,000 cells per well and incubated with conditioned medium at 37°C. After 4–6 hours, capillary network structures were imaged by phase‐contrast microscopy (Leica Microsystems) and the total number of interconnecting branches in each group counted using Image‐Pro software (Media Cybernetics, Rockville, MD, http://www.mediacy.com).

### Isolation of Exosomes

Conditioned medium was collected from each MSC type after culture in DMEM with 10% exosome‐depleted FBS for 48 hours. Media were passed through a 0.22‐μm filter to remove cell debris and incubated for 24 hours on ice with 0.5 volume of total exosome isolation reagent (Thermo Fisher Scientific). The mixture was centrifuged at 10,000*g* for 1 hour and the supernatant was discarded. Exosome pellets were resuspended in serum‐free medium and prepared for protein or RNA analysis.

### Transmission Electron Microscopy

To investigate exosome morphology, 3 μl of exosome pellets were layered onto 200‐mesh copper electron microscopy grids for 5 minutes at room temperature and stained with uranyl acetate 14. After three washes with PBS, exosomes were observed under a transmission electron microscope (H7500 TEM, Hitachi, Tokyo, Japan, http://www.hitachi.com), and micrographs were taken to quantify the size of the exosomes.

### PKH‐26 Labeling and Staining

To label the exosomes, 4 µl of PKH‐26, (Sigma‐Aldrich) was added to 1 ml of diluent containing 30 μg of MSC‐derived exosomes and incubated at room temperature for 15 minutes. Then, 1 ml of 5% BSA was added to stop the reaction, and the suspension was centrifuged at 400*g* for 10 minutes. The supernatant was discarded, and the cell pellets were resuspended in 10 ml of PBS and centrifuged at 400*g* for 5 minutes. The cell pellets were washed twice with 10 ml of complete medium to remove any unbound dye.

### Exosome Inhibition

EnMSCs were cultured to 90% confluence in complete medium. The media were replaced with DMEM containing 2% exosome‐depleted FBS, and the cells were conditioned with or without 20 μM GW4869 (Sigma‐Aldrich) for an additional 48 hours. Conditioned media were collected for in vitro studies, and EnMSCs were prepared for in vivo studies.

### Exosomal miR Extraction and Microarray

Total RNA was extracted from exosomes using a total exosome RNA isolation kit (Thermo Fisher Scientific), according to the manufacturer's instructions. RNA pellets were dissolved in RNase‐free water, and RNA content was determined using an Agilent 2100 Bioanalyzer (Agilent Technologies, Santa Clara, CA, http://www.genomics.agilent.com). RNA samples of 100 ng were used for miR microarrays. RNA was labeled using a miRCURY Hy3/Hy5 Power labeling kit (Exiqon, Vedbaek, Denmark, http://www.exiqon.com), hybridized on a miRCURY LNA Array, version 18.0 (Exiqon), and scanned using an Axon GenePix 4000B microarray scanner (Molecular Devices, Sunnyvale, CA, http://www.moleculardevices.com). Expression data were analyzed using GenePix Pro 6.0 software (Axon, Jakarta, Indonesia, http://www.axoninstruments.biz).

### Lentiviral Vector Construction and Infection

Recombinant lentivirus was constructed by Genechem (Shanghai, China, http://www.genechem.com). For viral infection of EnMSCs, CMs, and HUVECs, the cells were seeded at a density of 1 × 10^5^ cells per well in a six‐well plate and infected with lentiviral vectors in the presence of 10 μg/ml polybrene (EMD Millipore, Merck KGaA, Darmstadt, Germany, http://www.emdmillipore.com). After 12 hours of infection, the medium was replaced with growth medium. At 72 hours after infection, the efficiency of the infection was assessed as green fluorescent protein expression by fluorescence microcopy and precisely quantified by real‐time polymerase chain reaction (RT‐PCR).

### RT‐PCR Analysis

Total RNAs and miRs were extracted, miRs were reverse transcribed using a miDETECT A Track quantitative RT (qRT)‐PCR Kit (RiboBio, Guangzhou, China, http://www.ribobio.com), and mRNAs were reverse transcribed using a PrimeScript Stand cDNA Synthesis Kit (Takara Bio Inc., Kusatsu, Japan, http://www.takara-bio.com). The mRNA primers were selected using Primer3 Input online software (available at: http://primer3.ut.ee/) as shown in supplemental online Table 1. The primers of miRs were designed using miRBase online software (available at: http://www.mirbase.org/) as shown in supplemental online Table 2. RT‐PCRs were conducted using SYBR green reaction mixture in the Step One Plus Real‐Time PCR System (ABI PRISM 7900HT; Thermo Fisher Scientific). The PCR conditions were 95°C for 10 minutes and 40 cycles at 95°C for 30 seconds, 60°C for 30 seconds, and 72°C for 1 minute. We used β‐actin as the internal control for normalization and calculated the relative expression of mRNA using a (ΔCt) value method. Relative expression of miRs were also evaluated by a ΔCt method and normalized to U6.

### Western Blotting

In brief, cells and exosomes were rinsed with PBS and lysed in RIPA buffer (50 mM TrisHCl, pH 8, 150 mM NaCl, 1% NP‐40, 0.5% sodium deoxycholate, and 0.1% SDS; Beyotime Institute of Biotechnology, Haimen, China, http://www.beyotime.com) containing Protease Inhibitor Cocktail Set III, EDTA‐Free (1:200 dilution; Calbiochem; EMD Millipore) on ice for 30 minutes. Then, cell lysates were collected and centrifuged at 12,000 rpm for 30 minutes at 4°C to acquire extracts. Protein concentrations were assessed using a BCA Protein Assay Kit (Thermo Fisher Scientific). Next, 30 μg of protein was analyzed by SDS‐polyacrylamide gel electrophoresis and transferred to polyvinylidene difluoride membranes (EMD Millipore). After 1 hour of blocking with PBST containing 5% BSA at room temperature, primary antibodies, including anti‐Bcl‐2, Bcl‐2‐associated X protein (Bax), caspase‐3, phosphorylated‐Akt/total‐Akt, PTEN, vascular endothelial growth factor (VEGF), CD63, and β‐actin (Abcam) were diluted to 1:1,000 and incubated with the membrane overnight at 4°C. The membranes were washed with PBST and incubated with 1:3,000 secondary conjuncted with horseradish peroxidase (HRP) antibody for 1 hour at room temperature. The membranes were immersed in enhanced chemiluminescence solution and images acquired by digital development with β‐actin as control for normalization.

### Surgical Model of Myocardial Infarction and MSC Delivery

Rats (Sprague‐Dawley, male, 6–8 weeks old, weight, 200–250 g) were fed a standard laboratory diet. All animal research protocols met the Guide for the Care and Use of Laboratory Animals (NIH Publication no. 85‐23, revised 1996) and were approved by the Animal Care and Use Committee of Zhejiang Province Medical Institute. The MI model was established as described previously 15. In brief, MI was induced in anesthetized rats by permanent ligation of the left anterior descending coronary artery with 8‐0 silk suture. Successful coronary occlusion was confirmed by blanching of the myocardium distal to the coronary ligation. The rats were randomized into seven groups: control group (PBS treatment), BM group (BMMSC treatment), Ad group (AdMSC treatment), En group (EnMSC treatment), En‐Null group (treatment with EnMSCs infected with null vector), En‐miR‐21‐KD group (treatment with EnMSCs infected with miR‐21 knockdown vector), and En‐GW4869 group (EnMSCs preconditioned with 20 μM GW4869). At 30 minutes after ligation, 30 μl of cell suspension containing 1 × 10^6^ cells were injected around the MI border zone at five sites (2 × 10^5^ cells per site). The control groups received the same volume of PBS.

### Echocardiography

Echocardiography was implemented at baseline and 3, 28, and 56 days after surgery. The rats were anesthetized with 2% isoflurane in 100% O_2_ and placed in the supine position. Two‐dimensional and M‐model echocardiographic images (17.5 MHz transducer; Vevo 2100; VisualSonics, Toronto, ON, Canada, http://www.visualsonics.com) were obtained at the level of the papillary muscles in an investigator‐blinded manner. The left ventricular end‐systolic diameter (LVESD) and left ventricular end‐diastolic diameter (LVEDD) were measured for at least three separate cardiac cycles. The left ventricular end‐systolic volume (LVESV) and left ventricular end‐diastolic volume (LVEDV) were calculated as 7.0 × LVESD^3^/(2.4 + LVESD) and 7.0 × LVEDD^3^/(2.4 + LVEDD), respectively. The ejection fraction (EF) and fractional shortening (FS) were calculated as [(LVEDV − LVESV)/LVEDV] × 100 and [(LVEDD − LVESD)/LVEDD] × 100, respectively.

### Preparation of Tissue Samples

The rats were sacrificed at 3, 28, and 56 days after MI. The hearts were excised and cut into transverse slices at the ventricle midline between the atrioventricular groove and the apex. The hearts were dehydrated in 30% sucrose solution for 12–24 hours at 4°C, embedded in Tissue‐Tek O.C.T. compound (Sakura Finetek, Tokyo, Japan, http://www.sakura-finetek.com) and snap frozen on dry ice. Samples were cut at 7 μm thickness and preserved at −80°C.

### Immunohistochemistry

For immunohistochemistry, the heart tissue sections were fixed in 4% paraformaldehyde, permeabilized with 0.2% Triton X‐100, and blocked with PBST containing 5% BSA. The samples were incubated with anti‐α‐SMA antibody (Abcam) and anti‐CD31 antibody (Abcam) at 4°C overnight. After several washes with PBST, the samples were reacted with HRP‐conjugated secondary antibody for 1 hour at room temperature. Immunopositive reactions were developed using a polymer 3,3′‐diaminobenzidine and sections mounted on gelatin‐coated slides. Images were taken at four to five randomly selected high‐magnification fields in the border zone of the ischemic myocardium, and the average number of microvessels per visual field was calculated.

### Masson Trichrome Staining

At 28 and 56 days after MI, Masson trichrome staining was performed for infarct zone evaluation. In brief, sections from different groups were fixed with 4% paraformaldehyde, washed with PBS, and stained using a Masson trichrome kit (Maixin‐Bio, Fuzhou, China, http://www.maxim.com.cn). The infarct area was calculated by the sum of the endocardial and epicardial length of the infarct zone divided by the total length of the endocardial and epicardial left ventricle using Image‐Pro software (Media Cybernetics) and expressed as a percentage of infarct size.

### Statistical Analysis

Statistical analyses were calculated using SPSS, version 16.0, software (IBM Corp., Armonk, NY, http://www.ibm.com). One‐way analysis of variance tests were used to compare the differences between groups. Data are presented as the mean ± SD. Differences were considered statistically significant at *p* < .05.

## Results

### FACS Profiles of BMMSCs, AdMSCs, and EnMSCs

After adherent culture, BMMSCs, AdMSCs, and EnMSCs all appeared as spindle‐shaped fibroblast‐like cells ( supplemental online Fig. 1A). Cell surface marker expression of MSCs from each source was determined by flow cytometry. All samples coexpressed equivalent mesenchymal surface markers, including CD29, CD90, CD105, CD166, and were negative for hematopoietic surface markers CD34 and CD45. The expression of CD117 was very low in all MSCs ( supplemental online Fig. 1B).

### Paracrine Effects of BMMSCs, AdMSCs, and EnMSCs In Vitro

**Figure 1:**
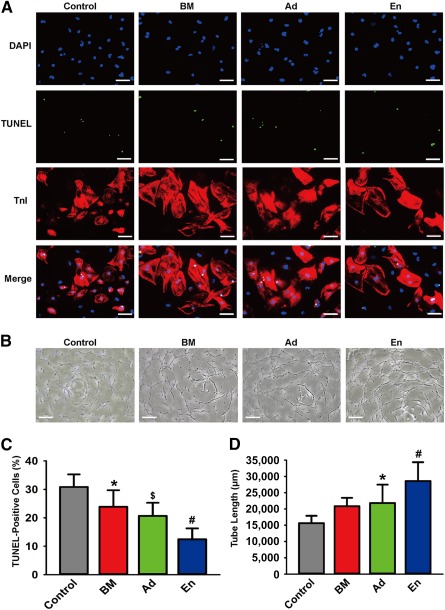
Comparison of paracrine effects of BMMSCs, AdMSCs, and EnMSCs in vitro. **(A):** Representive views of TUNEL‐positive CMs. Scale bars = 50 μm. **(B):** Representative views of tube formation of HUVECs. Scale bars = 100 μm. **(C):** Quantification of apoptotic CMs (*n* = 5 per group). Conditioned medium from EnMSCs induced the lowest rate of apoptotic CMs in all the groups. ∗, *p* < .05 versus control; $, *p* < .05 versus BM and control; #, *p* < .05 versus Ad, BM, and control. **(D):** Quantitative analysis of tube length (*n* = 5 per group). Conditioned medium generated from EnMSCs generated the longest tube extensions in all groups. ∗, *p* < .05 versus control; #, *p* < .05 versus Ad, BM, and control. Abbreviations: Ad, adipose; AdMSCs, adipose‐derived mesenchymal stem cells; BM, bone marrow; BMMSCs, bone marrow‐derived mesenchymal stem cells; CMs, cardiomyocytes; DAPI, 4′,6‐diamidino‐2‐phenylindole; En, endometrium; EnMSCs, endometrium‐derived mesenchymal stem cells; HUVECs, human umbilical vein endothelial cells; TnI, troponin I; TUNEL, terminal deoxynucleotidyl transferase dUTP nick‐end labeling.

Paracrine actions can contribute significantly to the cardioprotective effects of MSC‐based therapy 16. To analyze paracrine activities, cardiac myocytes or HUVECs were exposed to conditioned medium from each of the three types of MSCs, and the effects on apoptosis or angiogenesis, respectively, were determined. Apoptosis was monitored by TUNEL assay of CMs during exposure to 48 hours of simulated ischemia with or without conditioned medium. As shown in Figure 1A, 1C, CMs cocultured with conditioned medium from EnMSCs displayed significantly fewer apoptotic cells relative to all other groups (TUNEL‐positive ratio, EnMSCs, 12.44% ± 3.84%; BMMSCs, 23.85% ± 5.85%; AdMSCs, 20.68% ± 4.60%; and control, 30.86% ± 4.42%). The HUVEC tube length served as an in vitro surrogate for angiogenesis. The mean tube length from the EnMSC group (28,578 ± 5,770 μm) was markedly increased relative to that of all other groups (control, 15,628 ± 2,271 μm; BMMSCs, 20,840 ± 2,576 μm; AdMSCs, 21,805 ± 5,672 μm; Fig. 1B, 1D). These findings suggest that EnMSC‐derived conditioned medium confers enhanced cardioprotection and proangiogenic stimuli relative to either of the other MSC types.

### Paracrine Effects of BMMSCs, AdMSCs, and EnMSCs During MI

The role of paracrine action as a mechanism for MSC‐mediated therapy for myocardial infarction is well recognized. Therefore, the paracrine effects of each MSC treatment group were compared by quantifying apoptosis, neovascularization, infarct size, and cardiac function in parallel in a rat model of MI.

**Figure 2:**
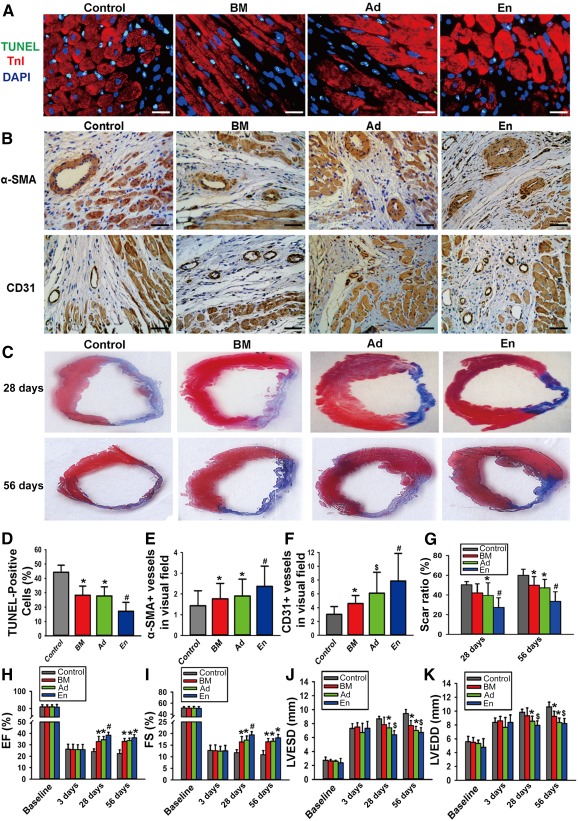
Comparison of paracrine potential of bone marrow‐derived mesenchymal stem cells (MSCs), adipose‐derived MSCs, and endometrium‐derived MSCs (EnMSCs) in vivo. **(A):** The infarcted rat hearts of all groups were excised 3 days after myocardial infarction (MI). Representative views of TUNEL‐positive nuclei. Scale bars = 10 μm. **(B):** Representative views of immunohistochemical staining with α‐SMA and CD31. Scale bars = 50 μm. **(C):** Representative Masson trichrome staining of heart to show infarct zone 28 and 56 days after MI. **(D):** Quantification of apoptotic nuclei (*n* = 5 per group). The lowest ratio of cardiac cell loss was in the EnMSC group. ∗, *p* < .05 versus control; #, *p* < .05 versus Ad, BM, and control. **(E):** Quantification of immunohistochemical staining of α‐SMA (*n* = 5 per group). Highest density of small arteries were present in the heart with EnMSC treatment. ∗, *p* < .05 versus control; #, *p* < .05 versus Ad, BM, and control. **(F):** Quantification of immunohistochemical staining of CD31 (*n* = 5 per group). Highest density of small veins were present in the heart with EnMSC treatment. ∗, *p* < .05 versus control; $, *p* < .05 versus BM and control; #, *p* < .05 versus Ad, BM, and control. **(G):** Quantification of infarct zone in heart tissue at both 28 and 56 days after MI. EnMSCs treatment induced smaller infarct area than other groups (*n* = 5 per group). ∗, *p* < .05 versus control; #, *p* < .05 versus Ad, BM, and control. **(H–K):** Quantitative analysis of echocardiography (*n* = 5 per group). Compared with other groups, the EnMSC group had better cardiac function indexes, including higher EF and FS and shorter LVESD and LVEDD. Quantification of EF, FS, LVESD, and LVEDD: ∗, *p* < .05 versus control; $, *p* < .05 versus BM and control; #, *p* < .05 versus Ad, BM, and control. Abbreviations: α‐SMA, α‐smooth muscle actin; Ad, adipose; BM, bone marrow; DAPI, 4′,6‐diamidino‐2‐phenylindole; EF, ejection fraction; En, endometrium; FS, fractional shortening; LVEDD, left ventricular end‐diastolic diameter; LVESD, left ventricular end‐systolic diameter; TnI, troponin I; TUNEL, terminal deoxynucleotidyl transferase dUTP nick‐end labeling.

TUNEL staining of cardiac cells in the border zones after MI revealed that hearts in the EnMSC group had the lowest apoptotic index compared with that of the other groups (17.06% ± 6.30% vs. control, 44.26% ± 4.88%; BMMSCs, 28.24% ± 6.40%; and AdMSCs, 27.78% ± 6.32%; Fig. 2A, 2D). Neovascularization, determined by α‐SMA/CD31 immunohistochemical staining, revealed that myocardial tissues in the EnMSC group had the highest density of microvessels relative to the other groups (Fig. 2B, 2E, 2F). Similarly, the infarct size as determined by Masson trichrome staining at 28 and 56 days after MI (Fig. 2C, 2G) was most reduced by EnMSC transplantation relative to all other groups (28 days after MI: EnMSCs, 27.34% ± 9.77%; control, 50.35% ± 3.23%; BMMSCs, 41.98% ± 9.32%; and AdMSCs, 39.55% ± 12.76%; and 56 days after MI: EnMSCs, 33.39% ± 9.86%; control, 59.90% ± 6.23%; BMMSCs, 49.93% ± 8.70%; and AdMSCs, 47.18% ± 8.63%). Similar trends were observed for cardiac function. Echocardiography revealed that transplantation of all MSCs reduced left ventricular dilation and preserved systolic function compared with the control group. However, the cardiac performance in the EnMSC treatment groups was significantly better than that of the BMMSC or AdMSC groups at both 28 and 56 days postoperatively (Fig. 2H–2K, supplemental online Fig. 2A). These results indicate the superior paracrine properties and therapeutic actions of EnMSCs relative to adipose‐ or BM‐derived cells.

### Validation of the Role of Exosomes in Paracrine Effects of EnMSCs

**Figure 3:**
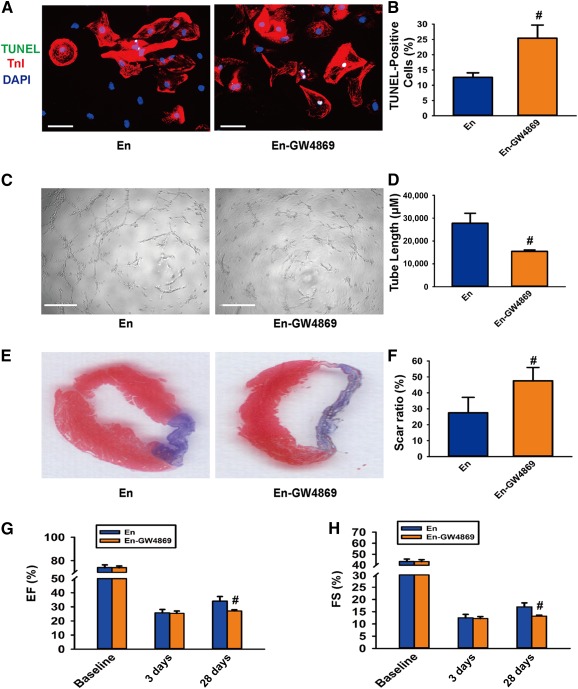
The role of exosomes was validated in paracrine effects of endometrium‐derived mesenchymal stem cells (EnMSCs). **(A):** Representive views of TUNEL‐positive cardiomyocytes (CMs). Scale bars = 50 μm. **(B):** Quantification of the apoptotic CMs (*n* = 5 per group). Conditioned medium from the En‐GW4869 group induced a higher rate of apoptotic CMs than in the En group. #, *p* < .05 versus En. **(C):** Representative views showing tube formation of human umbilical vein endothelial cells. Scale bars = 100 μm. **(D):** Quantification analysis of tube length (*n* = 5 per group). Conditioned medium from the En‐GW4869 group induced shorter tube extensions than in the EnMSC group. #, *p* < .05 versus En. **(E):** Representative Masson trichrome staining of heart tissue to show infarct zone at 28 days after myocardial infarction (MI). **(F):** Quantification of infarct zone in heart tissue at 28 days after MI (*n* = 5 per group). En‐GW4869 treatment induced a larger scar area than in the other groups. #, *p* < .05 versus En. **(G, H):** Quantitative analysis of echocardiography (*n* = 5 per group). GW4869 blocked cardiac function improvements induced by EnMSCs. #, *p* < .05 versus En. Abbreviations: DAPI, 4′,6‐diamidino‐2‐phenylindole; EF, ejection fraction; En, endometrium; En‐GW4869, EnMSCs preconditioned with 20 mM GW4869; FS, fractional shortening; TnI, troponin I; TUNEL, terminal deoxynucleotidyl transferase dUTP nick‐end labeling.

If exosomes regulate the therapeutic effects of EnMSCs, inhibition of exosome release would be expected to abolish the cardioprotection. To verify this hypothesis, we treated EnMSCs with GW4869, a reversible blocker of neutral sphingomyelinase that inhibits exosome secretion 17, 18. Exosome production was inhibited by GW4869 in a dose‐dependent manner, with complete blockage at a concentration of 20 μM ( supplemental online Fig. 3A). We found that suppression of exosome secretion by GW4869 abrogated the antiapoptosis and proangiogenesis actions of EnMSCs in vitro as evidenced in either the apoptotic indexes of CMs under hypoxia or capillary structure formations by HUVECs in Matrigel (BD Biosciences; Fig. 3A–3D). In vivo, EnMSCs pretreated with GW4869 conferred no infarct size reduction or improvement of cardiac performance as indicated by echocardiography (Fig. 3E–3H, supplemental online Fig. 3B). These results confirm that secreted exosomes are essential for EnMSC‐mediated cardiac repair.

### Characterization and Internalization of Exosomes

**Figure 4:**
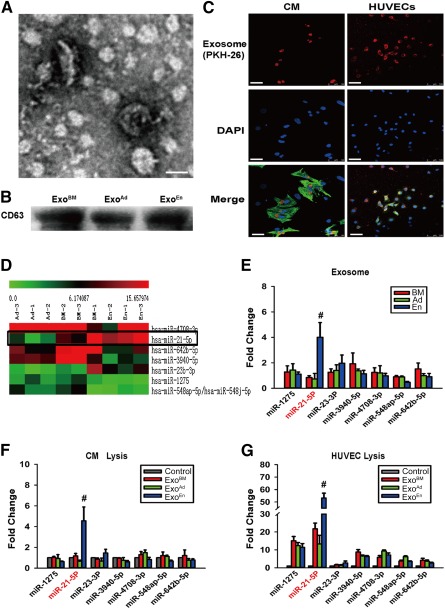
Mesenchymal stem cell (MSC)‐derived exosomes were characterized and taken up by CMs and HUVECs. Comparison of different expression of miRNAs in exosomes and recipient cells. **(A):** Shape and size of exosome. Scale bar = 100 nm. **(B):** Representative marker CD63 for exosome purity confirmation. **(C):** Cellular internalization of MSC‐derived exosomes into CMs and HUVECs. Scale bars = 50 μm. **(D):** View of exosomal miRNA array analysis. Expression of miR‐21 was highest in EnMSC exosomes. **(E):** Polymerase chain reaction identification of exosomal miRNA array. Level of miR‐21 was highest in EnMSC exosomes (*n* = 3 per group). #, *p* < .05 versus Ad and BM. **(F, G):** Levels of cellular miR‐21 were highest in both CMs and HUVECs with EnMSC exosome treatment (*n* = 3 per group). #, *p* < .05 versus Ad, BM, and control. Abbreviations: Ad, adipose; BM, bone marrow; CMs, cardiomyocytes; d, day; DAPI, 4′,6‐diamidino‐2‐phenylindole; En, endometrium; EnMSCs, endometrium‐derived mesenchymal stem cells; Exo, exosome; HUVECs, human umbilical vein endothelial cells; miR, microRNA.

To investigate the possible molecular mediators of MSC‐derived paracrine effects, we isolated exosomes from conditioned medium as described in Materials and Methods. Transmission electron microscopy, implemented as described previously 19, revealed the predicted shape and size of exosomes 20 (Fig. 4A). Immunoblotting also confirmed enrichment of the exosomal surface marker CD63 in purified exosomal fractions (Fig. 4B). To confirm cellular exosome uptake, exosome fractions from each conditioned medium were labeled with PKH26 and cocultured with CMs and HUVECs. After 6 hours, the cells were immunostained and analyzed by confocal laser microscopy to detect PKH26 fluorescence. As shown in Figure 4C, both CMs and HUVECs stained positively for PKH26, confirming exosomal uptake.

### Expression of miRs in Exosomes and Recipient Cells

We compared miR profiles in exosomes derived from each MSC group by microarray analyses as described in Materials and Methods. As shown in Figure 4D, exosomal miR profiles from EnMSCs were different from those of the other groups, in particular, miR‐1275, miR‐21‐5p, miR‐23‐3p, miR‐3940‐5p, miR‐4708‐3p, miR‐548ap‐5p, and miR‐642b‐5. qRT‐PCR validation confirmed these results and further revealed that the expression of miR‐21 was selectively increased more significantly in EnMSC exosomes relative to all other groups (Fig. 4E). Enhanced expression of miR‐21 selectively in the EnMSC group was also seen in CMs and HUVECs after 24 hours of coculture with each exosomal fraction (Fig. 4F, 4G).

### Analysis of miR‐21 Predicted Targets

**Figure 5:**
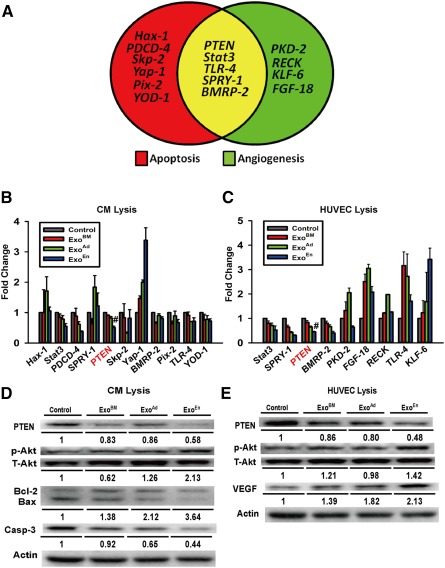
Predicted target genes of microRNA‐21 (miR‐21) were validated in CMs and HUVECs. **(A):** Target genes of miR‐21 involved in both apoptosis and angiogenesis. **(B, C):** Polymerase chain reaction identification of predicted target genes of miR‐21 in recipient cells and mRNA expression of PTEN decreased most sharply in both CMs and HUVECs after endometrium mesenchymal stem cell exosome incubation (*n* = 3 per group). #, *p* < .05 versus Ad, BM, and control. **(D, E):** Western blot identification for PTEN/Akt pathway activation after exosome incubation. Quantitation of PTEN, p‐Akt, Akt, Bcl‐2, Bax, caspase‐3, VEGF, and actin expression in CMs and HUVECs (*n* = 3). Abbreviations: Ad, adipose; Bax, Bcl‐2‐associated X protein; Bcl‐2, B‐cell CLL/lymphoma 2; BM, bone marrow; *BMRP‐2*, Bcl‐2 interacting mitochondrial ribosomal protein 2; Casp‐3, caspase‐3; CMs, cardiomyocytes; *FGF‐18*, fibroblast growth factor 18; *Hax‐1*, HS1‐associated protein X‐1; HUVECs, human umbilical vein endothelial cells; *KLF‐6*, Kruppel‐like factor 6; p‐Akt, phosphorylated Akt; *PDCD‐4*, programmed cell death 4; *Pix‐2*, POC1 centriolar protein A; *PKD‐2*, polycystin 2, transient receptor potential cation channel; *PTEN*, phosphatase and tensin homolog; *RECK*, reversion‐inducing cysteine‐rich protein with Kazal motif; *Skp‐2*, S‐phase kinase‐associated protein 2; *SPRY‐1*, sprouty homolog 1; *Stat3*, signal transducer and activator of transcription 3; T‐Akt, total Akt; *TLR‐4*, toll‐like receptor 4; VEGF, vascular endothelial growth factor; *Yap‐1*, Yes‐associated protein 1; *YOD‐1*, YOD1 deubiquitinase.

Target genes were predicted by gen ontology analysis, and genes were classified into three groups: molecular function, biological process, and cellular component. Apoptosis‐ and angiogenesis‐related miR‐21 targets were selected for further identification. Putative miR‐21 apoptotic target genes included HS1‐associated protein X‐1 (*Hax‐1*), programmed cell death 4 (*PDCD‐4*), S‐phase kinase‐associated protein 2 (*Skp‐2*), Yes‐associated protein 1 (*Yap‐1*), POC1 centriolar protein A (*Pix‐2*), YOD1 deubiquitinase (*YOD‐1*), signal transducer and activator of transcription 3 (*Stat3*), phosphatase and tensin homolog (*PTEN*), toll‐like receptor 4 (*TLR‐4*), sprouty homolog 1 (*SPRY‐1*), and Bcl‐2 interacting mitochondrial ribosomal protein 2 (*BMRP‐2*). Angiogenesis target genes included polycystin 2, transient receptor potential cation channel (*PKD‐2*), reversion‐inducing cysteine‐rich protein with Kazal motif (*RECK*), Kruppel‐like factor 6 (*KLF‐6*), fibroblast growth factor 18 (*FGF‐18*), *Stat3*, *PTEN*, *TLR‐4*, *SPRY‐1*, and *BMRP‐2*. Target gene primers were selected using Primer3 Input online software (available at: http://primer3.ut.ee/), as listed in supplemental online Table 1. To identify possible apoptotic target genes, CMs were cocultured with exosome fractions under hypoxia (1% O_2_; 5% CO_2_), and RNA and protein were extracted after 48 hours. For angiogenesis, HUVECs were incubated with exosomes and RNA and protein extracted after 24 hours. Putative target genes were identified by qRT‐PCR. Both assays revealed that *PTEN* mRNA was uniquely decreased by treatment with EnMSC exosomes (Fig. 5A–5C). Western blots further confirmed that PTEN protein was most attenuated in recipient cells incubated with EnMSC exosomes, and this coincided with enhanced Akt phosphorylation and Bcl‐2 expression in CMs and VEGF expression in HUVECs (Fig. 5D, 5E).

### Further Validation of miR‐21 in Recipient Cells

**Figure 6:**
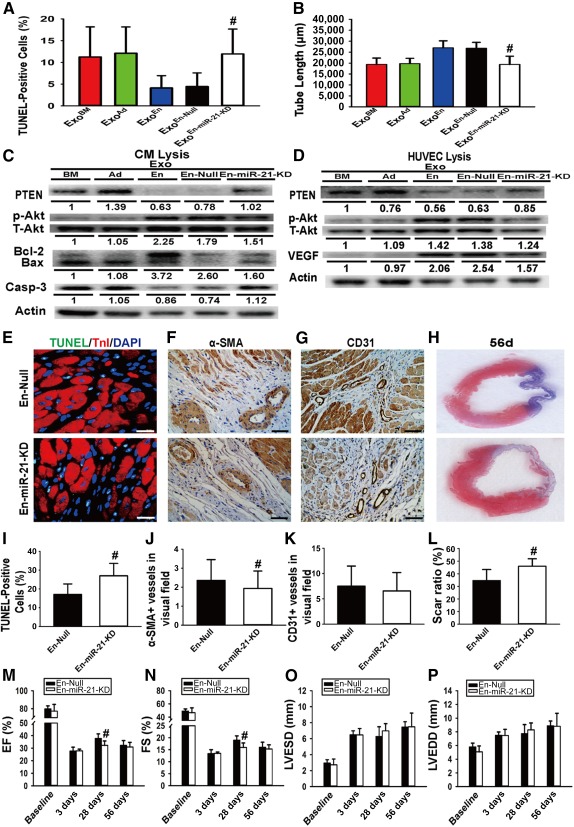
Exosomal miR‐21 validation of endometrium‐derived mesenchymal stem cells (EnMSCs) both in vitro and in vivo. CMs and HUVECs were incubated with exosomes secreted from bone marrow‐derived MSCs, adipose‐derived MSCs, EnMSCs, EnMSCs infected with null vector (EnMSC^Null^) and EnMSC infected with miR‐21 knockdown vector (EnMSC^miR‐21‐KD^) for in vitro study. EnMSC^Null^ and EnMSC^miR‐21‐KD^ were transplanted into infarcted rat hearts for the in vivo part of the study. **(A):** Quantification of the apoptotic CMs (*n* = 5 per group). Antiapoptosis effect in CMs was abolished after incubation of EnMSC^miR‐21‐KD^‐derived exosomes. #, *p* < .05 versus Exo^En^ and Exo^En‐Null^. **(B):** Quantitative analysis of tube length (*n* = 5 per group). Angiogenesis effect in HUVECs was abolished after incubation of EnMSC^miR‐21‐KD^‐derived exosomes. #, *p* < .05 versus Exo^En^ and Exo^En‐Null^. **(C, D):** Western blot identification for PTEN/Akt pathway activation in recipient cells after incubation of different exosomes. Quantitation of PTEN, p‐Akt, Akt, Bcl‐2, Bax, caspase‐3, VEGF, and actin expression in CMs and HUVECs (*n* = 3). **(E):** The infarcted rat hearts of all groups were excised at 3 days after myocardial infarction (MI). Representative views of TUNEL‐positive nuclei. Scale bars = 10 μm. **(F):** Representative views of immunohistochemical staining with α‐SMA. Scale bars = 50 μm. **(G):** Representative views of immunohistochemical staining with CD31. Scale bars = 50 μm. **(H):** Representative Masson trichrome staining of heart to show infarct zone 56 days after MI. **(I):** Quantification of the apoptotic nuclei (*n* = 5 per group). Higher ratio of cardiac cell loss was found in the En‐miR‐21‐KD group. #, *p* < .05 versus En‐Null. **(J):** Quantification of immunohistochemical staining of α‐SMA (*n* = 5 per group). Fewer small arteries were present in the hearts with En‐miR‐21‐KD treatment. #, *p* < .05 versus En‐Null. **(K):** Quantification of immunohistochemical staining of CD31 (*n* = 5 per group). Relatively lower density of microvessels were present in the heart with En‐miR‐21‐KD treatment. **(L):** Quantification of infarct zone in heart tissue at 56 days after MI (*n* = 5 per group). En‐miR‐21‐KD treatment induced a larger infarct area than in the En‐Null groups. #, *p* < .05 versus En‐Null. **(M–P):** Quantitative analysis of echocardiography (*n* = 5 per group). Compared with the En‐Null group, the En‐miR‐21‐KD group had worse cardiac function indexes. #, *p* < .05 versus En‐Null. Abbreviations: α‐SMA; α‐smooth muscle actin; Ad, adipose; Bax, Bcl‐2‐associated X protein; Bcl‐2, B‐cell CLL/lymphoma 2; BM, bone marrow; Casp‐3, caspase‐3; CMs, cardiomyocytes; DAPI, 4′,6‐diamidino‐2‐phenylindole; En, endometrium; En‐miR‐21‐KD, EnMSCs infected with miR‐21 knockdown vector; En‐Null, EnMSCs infected with null vector; Exo, exosome; HUVECs, human umbilical vein endothelial cells; LVEDD, left ventricular end‐diastolic diameter; LVESD, left ventricular end‐systolic diameter; miR‐21, microRNA‐21; p‐Akt, phosphorylated Akt; PTEN, phosphatase and tensin homolog; T‐Akt, total Akt; TnI, troponin I; TUNEL, terminal deoxynucleotidyl transferase dUTP nick‐end labeling; VEGF, vascular endothelial growth factor.

To validate a role for miR‐21 in EnMSC‐specific paracrine and cardioprotective effects, EnMSCs were transfected with a miR‐21 antagonist miR or negative control as described in Materials and Methods. For in vitro study, exosomes derived from EnMSC^miR‐21‐KD^, EnMSC^Null^, EnMSCs, BMMSCs, and AdMSCs were incubated with CMs or HUVECs under hypoxic or normoxic conditions, followed by apoptosis or angiogenesis assays, respectively. For in vivo validation, EnMSC^miR‐21‐KD^ and EnMSC^Null^, respectively, were delivered into infarcted rat hearts. PTEN and its downstream targets were simultaneously evaluated by Western blotting. In parallel experiments, CMs and HUVECs were transfected directly with miR‐21 or a negative control and subjected to hypoxic or normoxic incubations. As shown in Figure 6, significantly higher apoptotic indexes were observed in CMs incubated with exosomes from EnMSC^miR‐21‐KD^ than CMs cocultured with EnMSC^Null^ and EnMSC‐derived exosomes, and HUVECs incubated with exosomes of EnMSC^miR‐21‐KD^ had a lower capillary density (Fig. 6A, 6B, supplemental online Fig. 4C, 4D). The efficiencies of miR‐21 knockdown in both EnMSCs and exosomes were quantified ( supplemental online Fig. 4A, 4B). Western blots confirmed that suppression of miR‐21 in exosomes conferred upregulation of PTEN, inactivation of Akt phosphorylation, and reduced expression of Bcl‐2 and VEGF in recipient cells (Fig. 6C, 6D). Moreover, the results shown in Figure 6 confirmed the absence of therapeutic enhancement in the EnMSC^miR‐21‐KD^ treatment group, including the loss of antiapoptosis and cardioprotective properties (Fig. 6E, 6I), loss of proangiogenic actions (Fig. 6F, 6G, 6J, 6K), absence of a reduction in infarct size (Fig. 6H, 6L), and no improvement in cardiac function (Fig. 6M–6P, supplemental online Fig. 5D). We attributed these effects to downregulation of miR‐21 and upregulation of PTEN in heart tissues of these treatment groups ( supplemental online Fig. 5A–5C). Compared with the null groups, CMs transfected with miR‐21 inhibitor displayed higher apoptotic indexes after hypoxia incubation, but miR‐21 KD HUVECs had lower microvessel densities ( supplemental online Fig. 6A, 6B, 6D, 6E). High efficiencies of miR‐21 knockdown by our procedures were confirmed in both CMs and HUVECs ( supplemental online Fig. 6C, 6F). Western blotting confirmed that blockage of miR‐21 conferred upregulation of PTEN, inactivation of Akt phosphorylation, and reduced expression of Bcl‐2 and VEGF in recipient cells ( supplemental online Fig. 6G, 6H).

## Discussion

**Figure 7:**
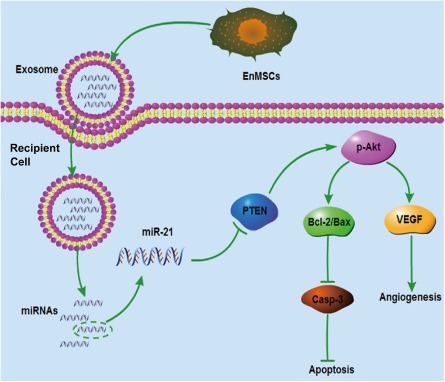
Schematic of superior cardioprotection, including antiapoptosis and angiogenesis, mediated by exosomal miR‐21 secreted by EnMSCs. Abbreviations: Bax, Bcl‐2‐associated X protein; Bcl‐2, B‐cell CLL/lymphoma 2; Casp‐3, caspase‐3; EnMSCs, endometrium‐derived mesenchymal stem cells; miR‐21, microRNA‐21; p‐Akt, phosphorylated Akt; PTEN, phosphatase and tensin homolog; VEFG, vascular endothelial growth factor.

Our results suggest that MSCs derived from the endometrium have greater therapeutic potential compared with cells derived from bone marrow or adipose tissue, the two most common sources of therapeutic MSCs 21, 22. We report stronger paracrine effects of EnMSC on cellular apoptosis and angiogenesis parameters in vitro and in vivo and greater myocardial salvage and enhanced cardiac function after MI in the EnMSC‐treatment groups. The enhanced paracrine actions and therapy with EnMSCs might be mediated by higher exosomal expression and cellular delivery of miR‐21 with downstream effects on the target PTEN‐Akt survival pathway (Fig. 7).

Clinical trials of MSCs derived from bone marrow or adipose tissue have demonstrated improved cardiac function that is probably mediated primarily by paracrine actions of secreted cytokines rather than by cardiac regeneration or myogenic differentiation 23, 24. However, bone marrow and adipose tissue might not be optimal cell sources for practical and functional reasons 20, 25. EnMSCs have several advantages, including simple, noninvasive isolation procedures, embryonic‐like properties, consistent quality, and improved function 7. Our present findings are the first to directly demonstrate the possible superiority of EnMSCs over MSCs from other sources for cardiovascular anti‐MI therapy. The improved therapy is driven by enhanced paracrine effects of EnMSCs that confer both improved cardioprotection and angiogenesis, as demonstrated by both in vitro and in vivo analyses.

Exosomal mRNA and miRs could be key components for biological information exchange and cell‐to‐cell communication 26. Recent work has suggested that MSC‐derived exosomes and exosomal miRs might contribute to the pathogenesis of stroke 27, multiple myeloma 28, breast cancer 29, and myocardial infarction 30. We have described, for the first time, high levels of miR‐21 in exosomes from EnMSCs that might be principal effectors of the superior paracrine effects of EnMSCs relative to bone marrow and adipose tissue. We found that exosomal miR‐21 released from EnMSCs can be shuttled to recipient CMs and endothelial cells to regulate apoptosis and angiogenesis in CMs and HUVECs, respectively, and ameliorate cardiac function after MI. Knockdown of miR‐21 in exosomes abolished these actions, further supporting an important role for miR‐21. Although our studies have confirmed essential contributions of miR‐21 to cardioprotection by EnMSC‐derived exosomes, it might not be the exclusive mediator of such protection. As shown in Figure 4, we have also confirmed the enhanced expression of miR‐23 in EnMSC‐derived exosomes and EnMSC‐exosome pretreated recipient cells. It has been reported that miR‐23 can promote angiogenesis in cardiac endothelial cells by targeting Sprouty2 and Sema6A and thereby activating proangiogenic signaling 31, 32. Therefore, it is possible that miR‐23 contributes in a similar way to the cardioprotective effects described in the present study for EnMSCs and their derived exosomes.

It was recently reported that exosomal miR‐21 derived from induced pluripotent stem cells protected the ischemic myocardium from apoptosis 33 and stimulated angiogenesis in transfected bronchial epithelial cells 34. Furthermore, as a downstream target of miR‐21, PTEN has important roles in both apoptosis and angiogenesis by regulating Akt phosphorylation 35, 36. Regulation of PTEN by miR‐21 was established in previous work in carcinoma 37 and cardiovascular cells 38. Consistent with this, we found that stimulation of miR‐21 in EnMSC exosomes reduced expression of PTEN and increased phosphorylation of Akt in exosome‐treated target cells and that these effects were reversed by knockdown of miR‐21, providing a molecular mechanism for the enhanced paracrine and cardioprotective effects of EnMSCs.

Our study has important clinical implications. MSCs are potential off‐the‐shelf reagents that are immune‐privileged, without autologous restrictions, and with wide application. The safety and feasibility of MSC therapy for ischemic myocardial disease has been well documented in both preclinical and early‐phase clinical trials, but improvements in myocardial function have been modest 23, 39 40 41 42. Thus, a medical need exists for new methods to improve therapy by MSCs. Our results demonstrate for the first time that EnMSCs confer significantly improved therapeutic benefit in the context of MI relative to BMMSCs and AdMSCs in parallel assays. The results support the use of EnMSCs as a preferential alternative to these other more commonly used cells, perhaps in combination with hypoxic preconditioning 43, for enhanced clinical treatment of heart disease.

## Conclusion

We have described the superior properties of EnMSCs relative to AdMSCs or BMMSCs for the treatment of myocardial infarction. EnMSCs conferred enhanced myocardium salvage and microvessel regeneration that was associated with significant improvement of cardiac function after MI. The possible mechanism for enhanced paracrine activity and therapy by EnMSC treatment might involve elevated expression and cellular delivery of exosomal miR‐21, with corresponding downstream effects on PTEN and Akt. Further investigations and clinical trials are essential to confirm EnMSCs as the preferred MSC source for myocardial regenerative medicine. Our study was limited to only three sources of MSCs. Thus, it is possible that MSCs from other early‐stage tissue sources, including placenta, fetus, and embryo, might have similar or superior properties to EnMSCs.

## Author Contributions

K.W.: conception and design, collection and/or assembly of data, data analysis and interpretation, manuscript writing; Z.J.: collection and/or assembly of data, data analysis and interpretation; K.A.W.: conception and design, data analysis and interpretation; J.C.: data analysis and interpretation, manuscript writing; H.H., Y.Z., L.W., Y.W., C.N., and Q.L.: collection and/or assembly of data; J.Z.: collection and/or assembly of data, data analysis and interpretation; Z.Z.: data analysis and interpretation, manuscript writing; C.X: provision of study material or patients; L.Z.: data analysis and interpretation, manuscript writing; R.W., W.Z., and H.Y.: data analysis and interpretation, manuscript writing; X.H. and J.W.: conception and design, data analysis and interpretation, provision of study material or patients, financial support, final approval of manuscript.

## Disclosure of Potential Conflicts of Interest

K.A.W. is founder, president, and CEO of Integene International LLC, a gene therapy company based in Miami, FL. The other authors indicated no potential conflicts of interest.

## Supporting information

Supporting Information

## References

[1] Dauwe DF , Janssens SP. Stem cell therapy for the treatment of myocardial infarction. Curr Pharm Des 2011;17:3328–3340. 21919877 10.2174/138161211797904208

[2] MacDonald DJ , Luo J , Saito T et al. Persistence of marrow stromal cells implanted into acutely infarcted myocardium: Observations in a xenotransplant model. J Thorac Cardiovasc Surg 2005;130:1114–1121. 16214528 10.1016/j.jtcvs.2005.04.033

[3] Atoui R , Shum‐Tim D , Chiu RC. Myocardial regenerative therapy: Immunologic basis for the potential “universal donor cells. Ann Thorac Surg 2008;86:327–334. 18573459 10.1016/j.athoracsur.2008.03.038

[4] Atoui R , Asenjo JF , Duong M et al. Marrow stromal cells as universal donor cells for myocardial regenerative therapy: Their unique immune tolerance. Ann Thorac Surg 2008;85:571–579. 18222266 10.1016/j.athoracsur.2007.10.034

[5] Rasmussen JG , Frøbert O , Holst‐Hansen C et al. Comparison of human adipose‐derived stem cells and bone marrow‐derived stem cells in a myocardial infarction model. Cell Transplant 2014;23:195–206. 23211469 10.3727/096368912X659871

[6] Meng X , Ichim TE , Zhong J et al. Endometrial regenerative cells: A novel stem cell population. J Transl Med 2007;5:57. 18005405 10.1186/1479-5876-5-57 PMC2212625

[7] Jiang Z , Hu X , Yu H et al. Human endometrial stem cells confer enhanced myocardial salvage and regeneration by paracrine mechanisms. J Cell Mol Med 2013;17:1247–1260. 23837896 10.1111/jcmm.12100 PMC3843975

[8] Zhang Z , Wang JA , Xu Y et al. Menstrual blood derived mesenchymal cells ameliorate cardiac fibrosis via inhibition of endothelial to mesenchymal transition in myocardial infarction. Int J Cardiol 2013;168:1711–1714. 23608402 10.1016/j.ijcard.2013.03.126

[9] Stoorvogel W , Kleijmeer MJ , Geuze HJ et al. The biogenesis and functions of exosomes. Traffic 2002;3:321–330. 11967126 10.1034/j.1600-0854.2002.30502.x

[10] Ge R , Tan E , Sharghi‐Namini S et al. Exosomes in cancer microenvironment and beyond: Have we overlooked these extracellular messengers?. Cancer Microenviron 2012;5:323–332. 22585423 10.1007/s12307-012-0110-2 PMC3460057

[11] Valadi H , Ekström K , Bossios A et al. Exosome‐mediated transfer of mRNAs and microRNAs is a novel mechanism of genetic exchange between cells. Nat Cell Biol 2007;9:654–659. 17486113 10.1038/ncb1596

[12] Arslan F , Lai RC , Smeets MB et al. Mesenchymal stem cell‐derived exosomes increase ATP levels, decrease oxidative stress and activate PI3K/Akt pathway to enhance myocardial viability and prevent adverse remodeling after myocardial ischemia/reperfusion injury. Stem Cell Res (Amst) 2013;10:301–312. 10.1016/j.scr.2013.01.002 23399448

[13] Simpson P , Savion S. Differentiation of rat myocytes in single cell cultures with and without proliferating nonmyocardial cells: Cross‐striations, ultrastructure, and chronotropic response to isoproterenol. Circ Res 1982;50:101–116. 7053872 10.1161/01.res.50.1.101

[14] Hu G , Yao H , Chaudhuri AD et al. Exosome‐mediated shuttling of microRNA‐29 regulates HIV Tat and morphine‐mediated neuronal dysfunction. Cell Death Dis 2012;3:e381. 22932723 10.1038/cddis.2012.114 PMC3434655

[15] Hu X , Yu SP , Fraser JL et al. Transplantation of hypoxia‐preconditioned mesenchymal stem cells improves infarcted heart function via enhanced survival of implanted cells and angiogenesis. J Thorac Cardiovasc Surg 2008;135:799–808. 18374759 10.1016/j.jtcvs.2007.07.071

[16] Gnecchi M , He H , Liang OD et al. Paracrine action accounts for marked protection of ischemic heart by Akt‐modified mesenchymal stem cells. Nat Med 2005;11:367–368. 15812508 10.1038/nm0405-367

[17] Kosaka N , Iguchi H , Yoshioka Y et al. Secretory mechanisms and intercellular transfer of microRNAs in living cells. J Biol Chem 2010;285:17442–17452. 20353945 10.1074/jbc.M110.107821 PMC2878508

[18] Trajkovic K , Hsu C , Chiantia S et al. Ceramide triggers budding of exosome vesicles into multivesicular endosomes. Science 2008;319:1244–1247. 18309083 10.1126/science.1153124

[19] Corcoran C , Rani S , O'Brien K et al. Docetaxel‐resistance in prostate cancer: Evaluating associated phenotypic changes and potential for resistance transfer via exosomes. PLoS One 2012;7:e50999. 23251413 10.1371/journal.pone.0050999 PMC3519481

[20] Heeschen C , Lehmann R , Honold J et al. Profoundly reduced neovascularization capacity of bone marrow mononuclear cells derived from patients with chronic ischemic heart disease. Circulation 2004;109:1615–1622. 15037527 10.1161/01.CIR.0000124476.32871.E3

[21] Wang Y , Li C , Cheng K et al. Activation of liver X receptor improves viability of adipose‐derived mesenchymal stem cells to attenuate myocardial ischemia injury through TLR4/NF‐κB and Keap‐1/Nrf‐2 signaling pathways. Antioxid Redox Signal 2014;21:2543–2557. 24915051 10.1089/ars.2013.5683 PMC4245883

[22] van der Bogt KE , Sheikh AY , Schrepfer S et al. Comparison of different adult stem cell types for treatment of myocardial ischemia. Circulation 2008;118 suppl:S121–S129. 18824743 10.1161/CIRCULATIONAHA.107.759480 PMC3657517

[23] Hare JM , Traverse JH , Henry TD et al. A randomized, double‐blind, placebo‐controlled, dose‐escalation study of intravenous adult human mesenchymal stem cells (prochymal) after acute myocardial infarction. J Am Coll Cardiol 2009;54:2277–2286. 19958962 10.1016/j.jacc.2009.06.055 PMC3580848

[24] Rodrigo SF , van Ramshorst J , Hoogslag GE et al. Intramyocardial injection of autologous bone marrow‐derived ex vivo expanded mesenchymal stem cells in acute myocardial infarction patients is feasible and safe up to 5 years of follow‐up. J Cardiovasc Transl Res 2013;6:816–825. 23982478 10.1007/s12265-013-9507-7 PMC3790917

[25] Behfar A , Yamada S , Crespo‐Diaz R et al. Guided cardiopoiesis enhances therapeutic benefit of bone marrow human mesenchymal stem cells in chronic myocardial infarction. J Am Coll Cardiol 2010;56:721–734. 20723802 10.1016/j.jacc.2010.03.066 PMC2932958

[26] Camussi G , Deregibus MC , Bruno S et al. Exosome/microvesicle‐mediated epigenetic reprogramming of cells. Am J Cancer Res 2011;1:98–110. 21969178 PMC3180104

[27] Xin H , Li Y , Liu Z et al. MiR‐133b promotes neural plasticity and functional recovery after treatment of stroke with multipotent mesenchymal stromal cells in rats via transfer of exosome‐enriched extracellular particles. STEM CELLS 2013;31:2737–2746. 23630198 10.1002/stem.1409 PMC3788061

[28] Roccaro AM , Sacco A , Maiso P et al. BM mesenchymal stromal cell‐derived exosomes facilitate multiple myeloma progression. J Clin Invest 2013;123:1542–1555. 23454749 10.1172/JCI66517 PMC3613927

[29] Ono M , Kosaka N , Tominaga N et al. Exosomes from bone marrow mesenchymal stem cells contain a microRNA that promotes dormancy in metastatic breast cancer cells. Sci Signal 2014;7:ra63. 24985346 10.1126/scisignal.2005231

[30] Kang K , Ma R , Cai W et al. Exosomes secreted from CXCR4 overexpressing mesenchymal stem cells promote cardioprotection via Akt signaling pathway following myocardial infarction. Stem Cells Int 2015;2015:659890. 26074976 10.1155/2015/659890 PMC4436515

[31] Dhanabal M , Wu F , Alvarez E et al. Recombinant semaphorin 6A‐1 ectodomain inhibits in vivo growth factor and tumor cell line‐induced angiogenesis. Cancer Biol Ther 2005;4:659–668. 15917651 10.4161/cbt.4.6.1733

[32] Impagnatiello MA , Weitzer S , Gannon G et al. Mammalian sprouty‐1 and ‐2 are membrane‐anchored phosphoprotein inhibitors of growth factor signaling in endothelial cells. J Cell Biol 2001;152:1087–1098. 11238463 10.1083/jcb.152.5.1087 PMC2198812

[33] Wang Y , Zhang L , Li Y et al. Exosomes/microvesicles from induced pluripotent stem cells deliver cardioprotective miRNAs and prevent cardiomyocyte apoptosis in the ischemic myocardium. Int J Cardiol 2015;192:61–69. 26000464 10.1016/j.ijcard.2015.05.020 PMC4469495

[34] Xu Y , Luo F , Liu Y et al. Exosomal miR‐21 derived from arsenite‐transformed human bronchial epithelial cells promotes cell proliferation associated with arsenite carcinogenesis. Arch Toxicol 2015;89:1071–1082. 24912785 10.1007/s00204-014-1291-x

[35] Liu Y , Nie H , Zhang K et al. A feedback regulatory loop between HIF‐1α and miR‐21 in response to hypoxia in cardiomyocytes. FEBS Lett 2014;588:3137–3146. 24983504 10.1016/j.febslet.2014.05.067

[36] Liu LZ , Li C , Chen Q et al. MiR‐21 induced angiogenesis through AKT and ERK activation and HIF‐1α expression. PLoS One 2011;6:e19139. 21544242 10.1371/journal.pone.0019139 PMC3081346

[37] Meng F , Henson R , Wehbe‐Janek H et al. MicroRNA‐21 regulates expression of the PTEN tumor suppressor gene in human hepatocellular cancer. Gastroenterology 2007;133:647–658. 17681183 10.1053/j.gastro.2007.05.022 PMC4285346

[38] Roy S , Khanna S , Hussain SR et al. MicroRNA expression in response to murine myocardial infarction: MiR‐21 regulates fibroblast metalloprotease‐2 via phosphatase and tensin homologue. Cardiovasc Res 2009;82:21–29. 19147652 10.1093/cvr/cvp015 PMC2652741

[39] Karantalis V , Hare JM. Use of mesenchymal stem cells for therapy of cardiac disease. Circ Res 2015;116:1413–1430. 25858066 10.1161/CIRCRESAHA.116.303614 PMC4429294

[40] Makkar RR , Smith RR , Cheng K et al. Intracoronary cardiosphere‐derived cells for heart regeneration after myocardial infarction (CADUCEUS): A prospective, randomised phase 1 trial. Lancet 2012;379:895–904. 22336189 10.1016/S0140-6736(12)60195-0 PMC4326004

[41] Mathiasen AB , Qayyum AA , Jørgensen E et al. Bone marrow‐derived mesenchymal stromal cell treatment in patients with severe ischaemic heart failure: A randomized placebo‐controlled trial (MSC‐HF trial). Eur Heart J 2015;36:1744–1753. 25926562 10.1093/eurheartj/ehv136

[42] Williams AR , Hare JM. Mesenchymal stem cells: Biology, pathophysiology, translational findings, and therapeutic implications for cardiac disease. Circ Res 2011;109:923–940. 21960725 10.1161/CIRCRESAHA.111.243147 PMC3604746

[43] Hu X , Wu R , Jiang Z et al. Leptin signaling is required for augmented therapeutic properties of mesenchymal stem cells conferred by hypoxia preconditioning. STEM CELLS 2014;32:2702–2713. 24989835 10.1002/stem.1784 PMC5096299

