# Supplementary material for: Enhanced Cardioprotection by Human Endometrium Mesenchymal Stem Cells Driven by Exosomal MicroRNA‐21
Source: Stem Cells Transl Med. 2016 Aug 29;6(1):209–22. doi: 10.5966/sctm.2015-0386 (PMC5442741; doi:10.5966/sctm.2015-0386)
Supplement: Supplementary file 1 — Supporting Information [file SCT3-6-209-s001.pdf]

Supplemental Information – Wang et al.

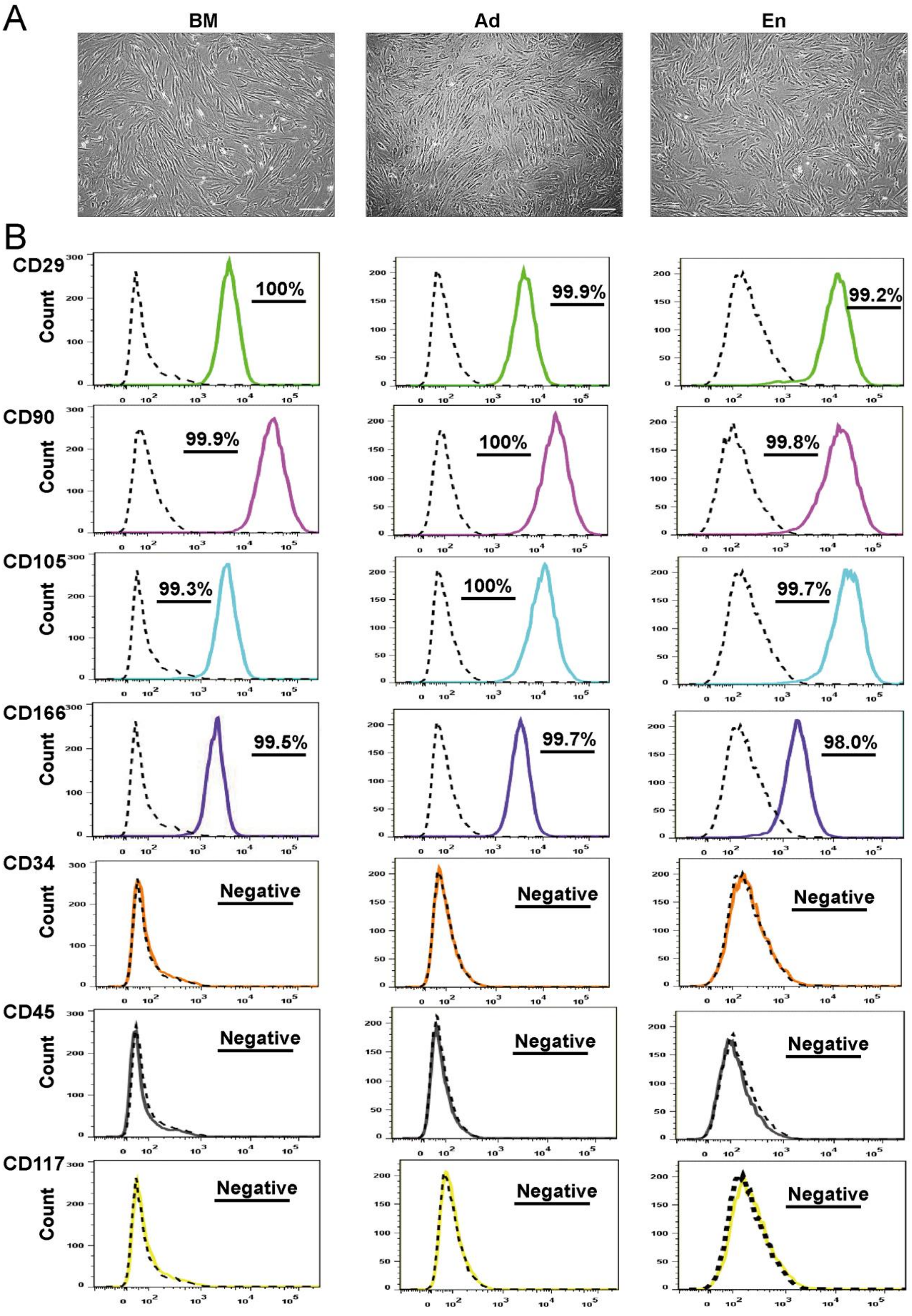

Fig S1. Morphologies and phenotypes of BMMSCs, AdMSCs and EnMSCs.

(A) Phase-contrast microscopic view of 100% confluent BMMSCs, AdMSCs and EnMSCs in culture medium, passage 6. Scale bar = 200μm (B) Cell surface markers of BMMSCs, AdMSCs and EnMSCs were analysed by flow cytometry, showing mesenchymal features.

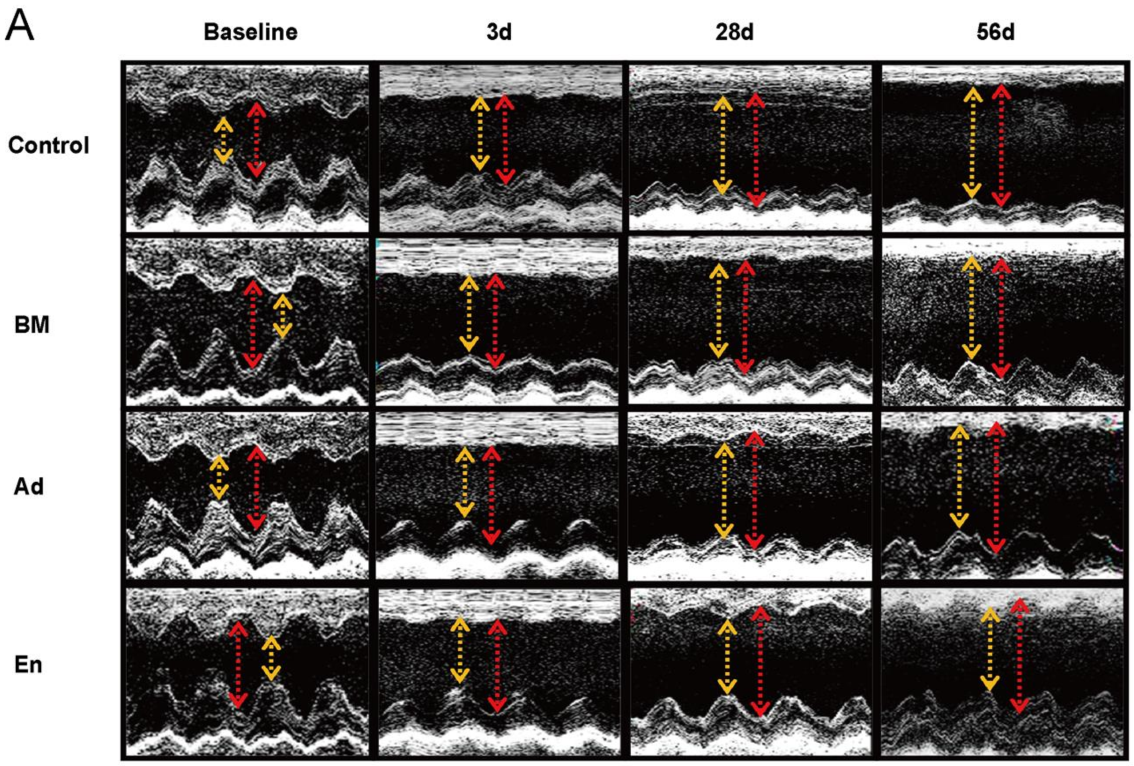

Fig S2. Representative M-mode echocardiographic images of MSCs treatment

(A) Representative M-mode echocardiographic images between Control, BMMSCs, AdMSCs and EnMSCs treatment at different time points.

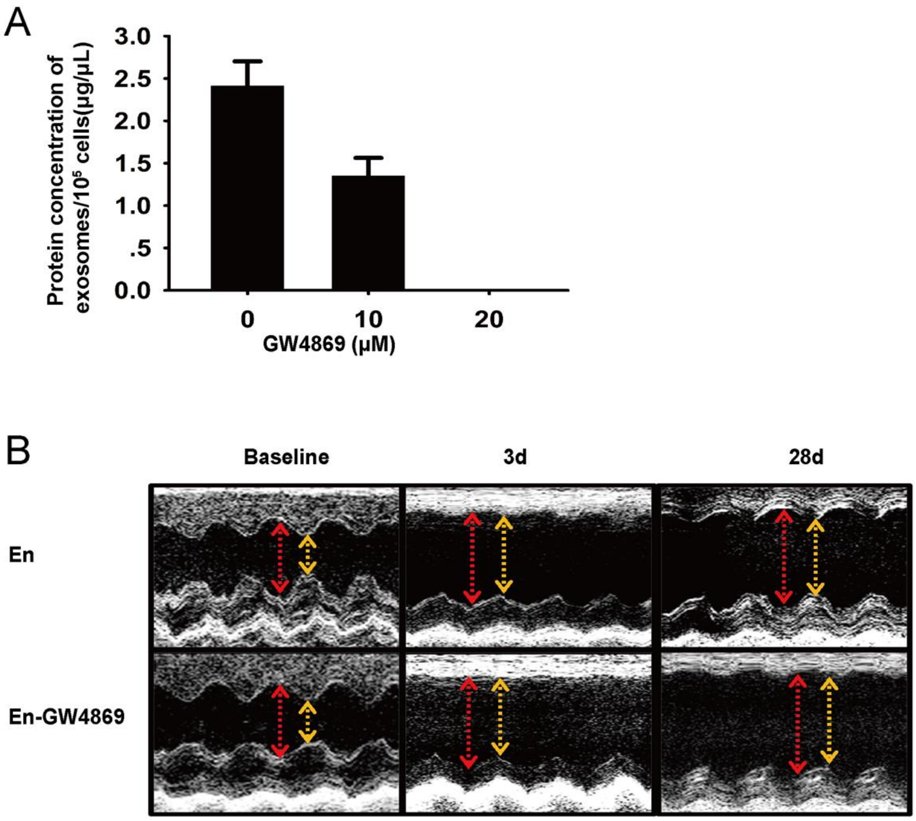

Fig S3. Suitable concentration of GW4869 in exosome inhibition and representative M-mode echocardiographic images.

(A) GW4869 inhibited exosome production in EnMSCs in a dose-dependent manner. (B) Representative M-mode echocardiographic images between En and En-GW4869 treatment at different time points.

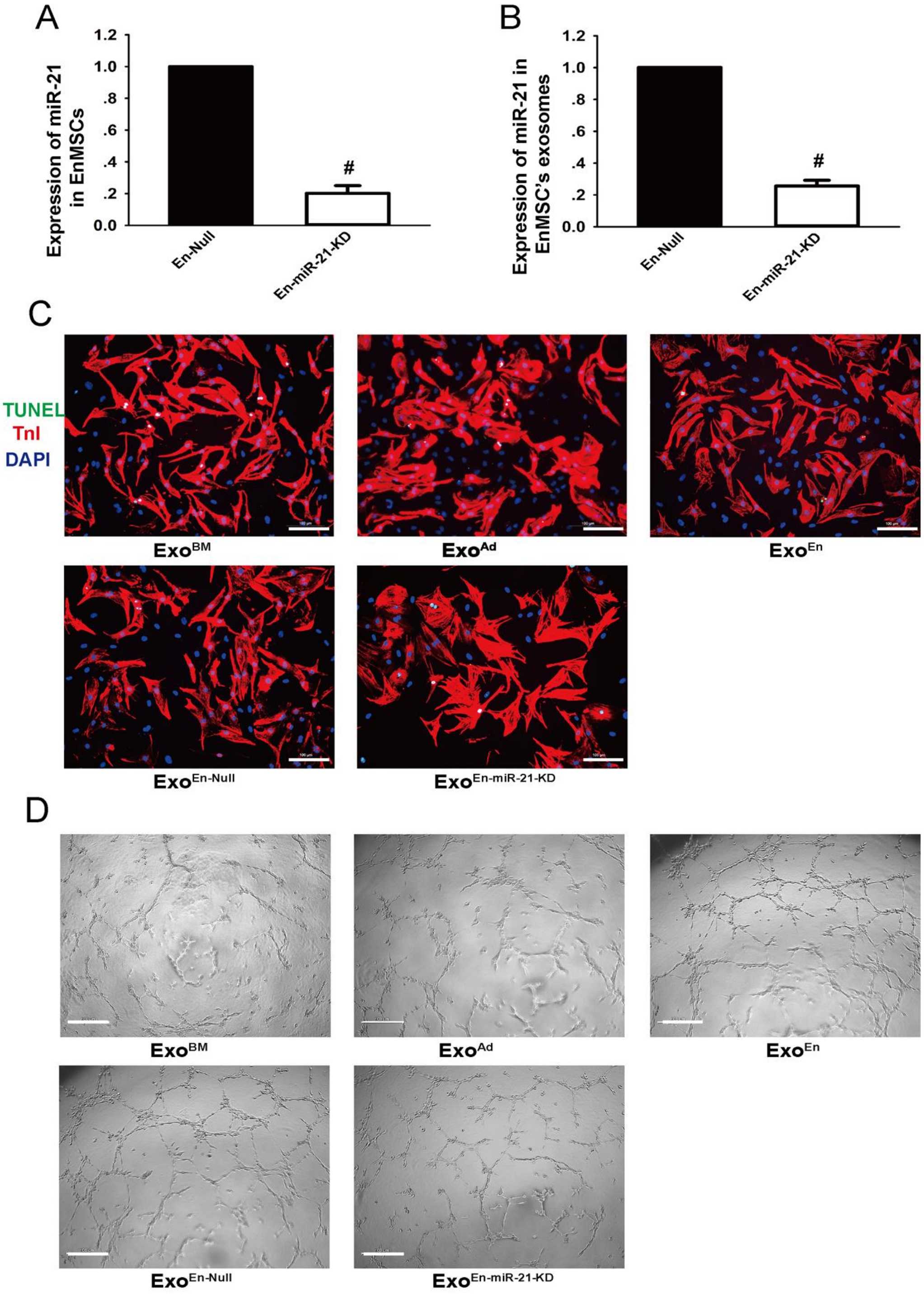
A
Expression of miR-21 in EnMSCs
1.2
1.0
.8
.6
.4
.2
0.0
#
En-Null
En-miR-21-KD
B
Expression of miR-21 in EnMSC's exosomes
1.2
1.0
.8
.6
.4
.2
0.0
#
En-Null
En-miR-21-KD
C
TUNEL
TnI
DAPI
Exo^BM
Exo^Ad
Exo^En
Exo^En-Null
Exo^En-miR-21-KD
D
Exo^BM
Exo^Ad
Exo^En
Exo^En-Null
Exo^En-miR-21-KD

Fig S4. Knockdown efficiencies of miR-21 KD in EnMSCs and representative images of in vitro study

(A) The levels of miR-21 in $EnMSC^{Null}$ and $EnMSC^{miR\text{-}21\text{-}KD}$ were determined by qRT-PCR (n=3/group). $^{\#}p<0.05$ versus En-Null. (B) The levels of miR-21 in exosomes secreted from $EnMSC^{Null}$ and $EnMSC^{miR\text{-}21\text{-}KD}$ were determined by qRT-PCR (n=3/group). $^{\#}p<0.05$ versus En-Null. (C) Representative pictures of TUNEL-positive CMs. Scale bar = 100μm. (D) Representative pictures showed tube formation of HUVECs. Scale bar = 100μm.

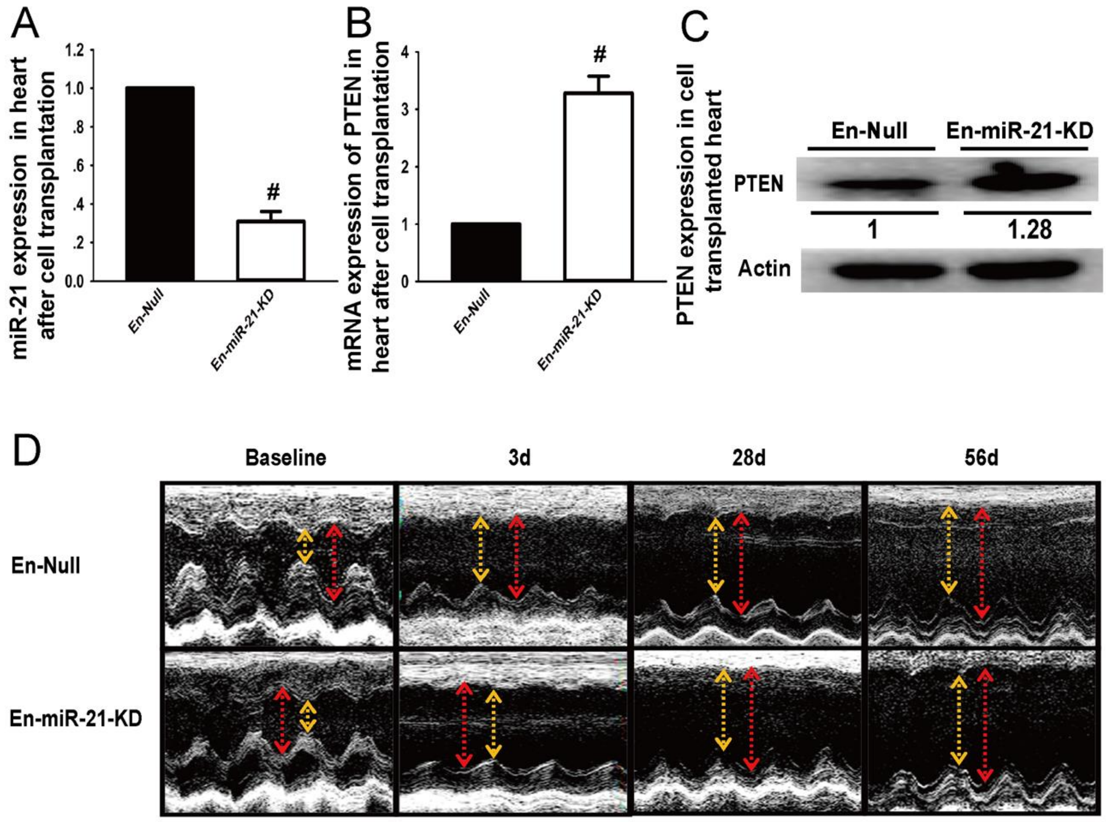

Fig S5. Validation of En-miR-21-KD transplantation in infarcted hearts and representative M-mode echocardiographic images

(A) PCR identification of miR-21 in heart tissue after cell transplantation (n=3/group). En-miR-21-KD treated heart displayed lower level of miR-21. $^{\#}p<0.05$ versus En-Null. (B) The mRNA expression of PTEN was determined by RT-PCR, and the level of that inreased more sharply in $EnMSC^{miR\text{-}21\text{-}KD}$ transplanted heart than $EnMSC^{Null}$ treatment group (n=3/group). $^{\#}p<0.05$ versus En-Null. (C) Western blot identification for PTEN expression and En-miR-21-KD treatment increased the level of PTEN in heart tissues. Quantitation of PTEN and Actin expession in heart tissues, respectively, n=3. (D) Representative M-mode echocardiographic images between En-Null and En-miR-21 KD treatment at different time points.

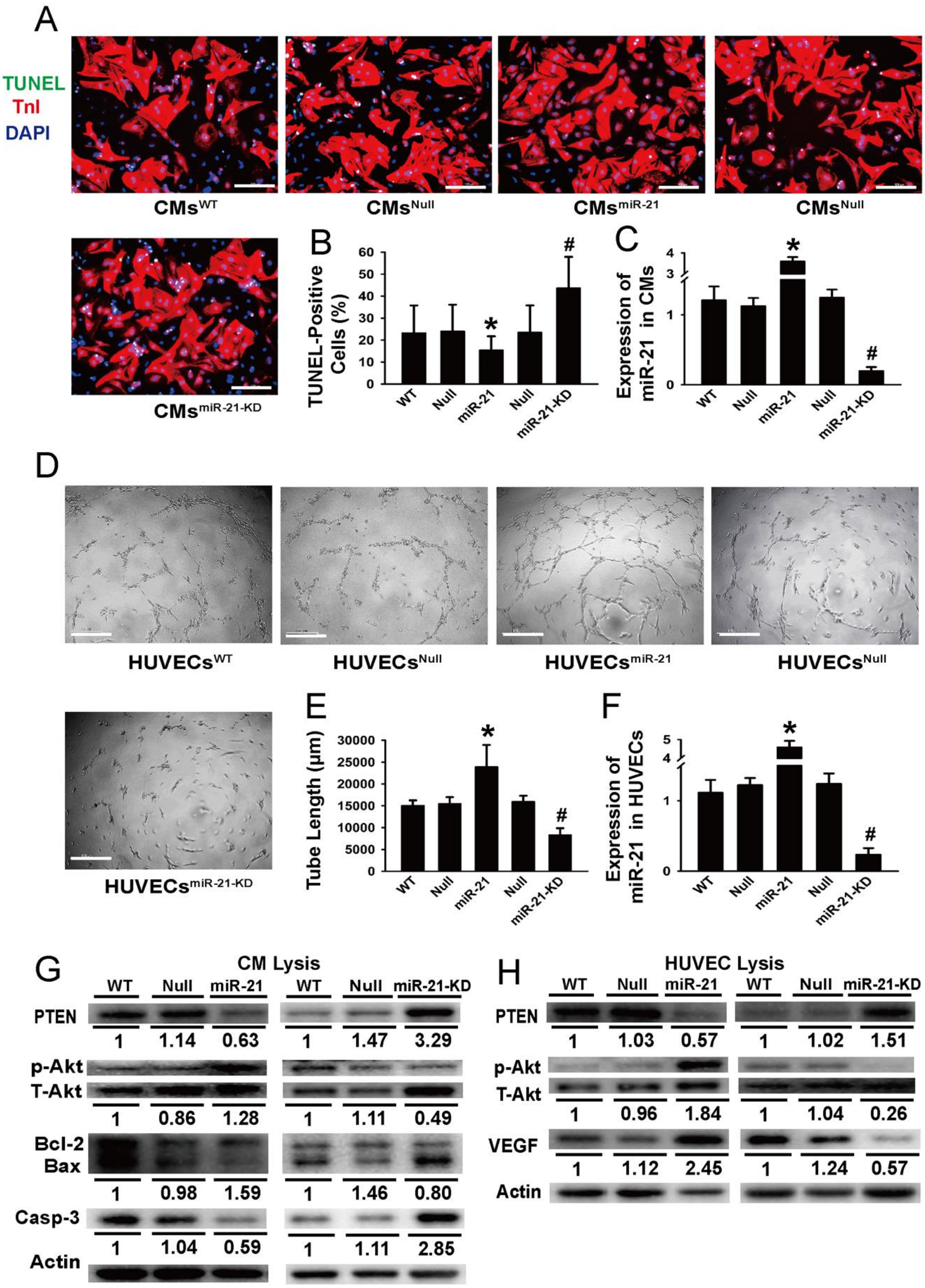
A
TUNEL
TnI
DAPI
CMs^WT
CMs^Null
CMs^miR-21
CMs^Null
CMs^miR-21-KD
B
TUNEL-Positive Cells (%)
WT
Null
miR-21
Null
miR-21-KD
C
Expression of miR-21 in CMs
D
HUVECs^WT
HUVECs^Null
HUVECs^miR-21
HUVECs^Null
HUVECs^miR-21-KD
E
Tube Length (μm)
F
Expression of miR-21 in HUVECs
G
CM Lysis
PTEN
1 1.14 0.63
1 1.47 3.29
p-Akt
T-Akt
1 0.86 1.28
1 1.11 0.49
Bcl-2
Bax
1 0.98 1.59
1 1.46 0.80
Casp-3
1 1.04 0.59
1 1.11 2.85
Actin
H
HUVEC Lysis
PTEN
1 1.03 0.57
1 1.02 1.51
p-Akt
T-Akt
1 0.96 1.84
1 1.04 0.26
VEGF
1 1.12 2.45
1 1.24 0.57
Actin

Fig S6. Direct miR-21 validation in recipient cells.

CMs and HUVECs were transfected with over-expressed miR-21 vector or miR-21-KD vector and Null vector. (A) Representative pictures of TUNEL-positive CMs. Scale bar = 100μm. (B) Quantification of the apoptotic CMs (n=5/group). Anti-apoptosis effect in CMs was abolished after tranfection of miR-21-KD. *p<0.05 versus WT and Null. #p<0.05 versus WT and Null. (C) The levels of miR-21 in WT, Null, miR-21-KD, and miR-21 overexpressed CMs were determined by qRT-PCR (n=3/group). *p<0.05 versus WT and Null. #p<0.05 versus WT and Null. (D) Representative pictures showed tube formation of HUVECs. Scale bar = 100μm. (E) Quantative analysis of tube length (n=5/group). Angiogenic effect in CMs was abolished after tranfection of miR-21-KD. *p<0.05 versus WT and Null. #p<0.05 versus WT and Null. (F) The levels of miR-21 in WT, Null, miR-21-KD, and miR-21 overexpressed HUVECs were determined by qRT-PCR (n=3/group). *p<0.05 versus WT and Null. #p<0.05 versus WT and Null. (G and H) Western blot identification for PTEN/Akt pathway activation in recepient cells after different exosomes incubation. Quantitation of PTEN, p-Akt, Akt, Bcl-2, Bax, Caspase-3, VEGF and Actin expession in CMs and HUVECs, respectively, n=3.

Supplemental Tables

Table S1 Primer Sequence of mRNA for Real-time RT-PCR

Gene Name	Primer sequence	Gene ID	Product size
Rat-Hax-1 F	GGTCTGGGTCCACTTCTTCA	NM 181627.2	156
Rat-Hax-1 R	ATGGGCTTCTTGATGGGTCA		
Rat-PDCD-4 F	AGGTTGCTAGATAGGCGGTC	XM 006231616.2	209
Rat-PDCD-4 R	GTCTTCTCAAACGCCGTCTC		
Rat-Skp-2 F	AGCTGCTCCTGGGAATCTTT	NM 001106416.2	209
Rat-Skp-2 R	CTGCTCCATAAACGACCGTG		
Rat-Yap-1 F	ACCCTCGTTTTGCCATGAAC	XM 006242493.2	195
Rat-Yap-1 R	CCTGCCGAAATAACTCCTGC		
Rat-Pix-2 F	TGTGTTTGCTTTTCCGGACC	NM 053624.2	229
Rat-Pix-2 R	ACATCCCCTTGGTGTAGTGG		
Rat-YOD-1 F	TTTGGGGAAGATGCAGGCTA	NM 001008889.1	226
Rat-YOD-1 R	GACACAGCATGCATCTCAGG		
Rat-Stat-3 F	TCAGTGAGAGCAGCAAGGAA	XM 006247259.2	226
Rat-Stat-3 R	TTTCCGAATGCCTCCTCCTT		
Rat-PTEN F	CCAGTCAGAGGCGCTATGTA	NM 031606.1	214
Rat-PTEN R	TACATGAGCTTGTCCTCCCG		
Rat-TLR-4 F	TTATCCAGAGCCGTTGGTGT	NM 019178.1	171
Rat-TLR-4 R	CCCACTCGAGGTAGGTGTTT		
Rat-SPRY-1 F	GCCGGCAGAGGTTAGACTAT	NM 001106427.1	235
Rat-SPRY-1 R	GCACAGTGAATCCAGAGCTG		

Rat-BMRP-2 F	TCCCAGAATCAAGAACGGCT	XM 006244952.2	246
Rat-BMRP-2 R	AATTGAGGGTGGGGTGGTAG		
Rat- Actin F	CAACCTTCTTGCAGCTCCTC	NM 031144.3	192
Rat- Actin R	ATACCCACCATCACACCCTG		
Hsa-PKD-2 F	GGAGACCAAGAACTGACCGA	XM 011532030.1	242
Hsa-PKD-2 R	ACTCGTCTCACCAGGACTTG		
Hsa-RECK F	GTACAGGTCTCCAGCAGTGT	XM 011518053.1	168
Hsa- RECK R	TTCAAGGCAGGAGAGTGAGG		
Hsa-KLF-6 F	AGAGCTGGAACGTTACCTCC	NM 001160125.1	221
Hsa-KLF-6 R	CGCTGCTGACATCTGAGTTC		
Hsa-FGF-18 F	AGTCAAGTCCGGATCAAGGG	NM 003862.2	161
Hsa- FGF-18 R	CCGGAGTACTTAGCCGACAT		
Hsa- Stat-3 F	AAAGCAGCAAAGAAGGAGGC	XM 011525146.1	234
Hsa- Stat-3 R	CTGGCCGACAATACTTTCCG		
Hsa- PTEN F	ACCGGCAGCATCAAATGTTT	NM 000314.6	176
Hsa- PTEN R	AGTTCCACCCCTTCCATCTG		
Hsa- TLR-4 F	ACCTCCCCTTCTCAACCAAG	NM 003266.3	150
Hsa- TLR-4 R	GGCTCTGATATGCCCCATCT		
Hsa- SPRY-1 F	CTGCTCCAATGACGACGAAG	XM 005262686.1	169
Hsa- SPRY-1 R	ATAACACCTCCTGCACAGCT		
Hsa- BMRP-2 F	CACCACTCAGTCCACCTCAT	XM 011511688.1	168
Hsa- BMRP-2 R	CTGCTGCCTCCATCATGTTC		
Hsa- Actin F	CGCAAAGACCTGTATGCCAA	XM 006715764.1	152
Hsa- Actin R	CACACAGAGTACTTGCGCTC		

Table S2 Primer Sequence of miRNAs for Real-time RT-PCR

Gene Name	Primer sequence
hsa-U6:	5’GATGACACGCAAATTCGTGAA 3’
hsa-miR-1275:	5’ GTG GGG GAG AGG CTG TC 3’
hsa-miR-21-5p:	5’ TAG CTT ATC AGA CTG ATG TTG A 3’
hsa-miR-23-3p:	5’ ATC ACA TTG CCA GGG ATT TCC 3’
hsa-miR—3940-5p:	5’ GTG GGT TGG GGC GGG CTC TG 3’
hsa-miR-4708-3p:	5’ AGC AAG GCG GCA TCT CTC TCT GAT 3’
hsa-miR-548ap-5p:	5’AAA AGT AAT TGC GGT CTT T 3’
hsa-miR-642b-5p:	5’ GGT TCC CTC TCC AAA TGT GTC T 3’
